# Digital sorting of complex tissues for cell type-specific gene expression profiles

**DOI:** 10.1186/1471-2105-14-89

**Published:** 2013-03-07

**Authors:** Yi Zhong, Ying-Wooi Wan, Kaifang Pang, Lionel ML Chow, Zhandong Liu

**Affiliations:** 1 Department of Pediatrics, Neurological Research Institute, Baylor College of Medicine, Houston, Texas, USA; 2 Computational and Integrative Biomedical Research Center, Baylor College of Medicine, Houston, Texas, USA; 3 Department of Obstetrics and Gynecology, Baylor College of Medicine, Houston, Texas, USA; 4 Cancer and Blood Diseases Institute, Cincinnati Children’s Hospital Medical Center, Cincinnati, Ohio, USA

## Abstract

**Background:**

Cellular heterogeneity is present in almost all gene expression profiles. However, transcriptome analysis of tissue specimens often ignores the cellular heterogeneity present in these samples. Standard deconvolution algorithms require prior knowledge of the cell type frequencies within a tissue or their in vitro expression profiles. Furthermore, these algorithms tend to report biased estimations.

**Results:**

Here, we describe a Digital Sorting Algorithm (DSA) for extracting cell-type specific gene expression profiles from mixed tissue samples that is unbiased and does not require prior knowledge of cell type frequencies.

**Conclusions:**

The results suggest that DSA is a specific and sensitivity algorithm in gene expression profile deconvolution and will be useful in studying individual cell types of complex tissues.

## Background

Cellular heterogeneity is present in nearly all biological specimens. When the genome-wide transcriptional profile of heterogeneous samples is measured under different physiological states, any observed differences are strongly confounded by differences in cell type compositions between samples [1-3]. Recent studies suggest that the microenvironment of a tissue may change under different physiological states and can contribute to the etiology of diverse diseases [4-10]. Consequently, to fully understand gene expression differences associated with different physiological states, deconvolution of tissue expression into the component expression profiles of each cell type is critically needed.

Fluorescence Activated Cell Sorting (FACS), Laser Capture Micro-dissection (LCM) and Translating Ribosome Affinity Purification (TRAP) have been used to physically separate defined cell types before gene expression analysis [2,11-13]. However, technical difficulties, such as limited availability of good surface markers, cell type specific promoters and transgenic models, have restricted the application of these techniques. Furthermore, the sorting process may introduce additional stress on cells and hence alter their gene expression profiles.

Unsupervised mixture models have been developed to solve the gene expression devolution problem. For example, a Latent Dirichlet Allocation (LDA) model trained with a variational expectation maximization framework was used to estimate the breast cancer cell gene expression profiles from heterogeneous tumor samples [14]. An alternative approach [15] is to first reduce the observed mixture using standard dimension reduction algorithm, such as principal components analysis (PCA) or independent components analysis (ICA), find a minimum-volume polytope with *k* vertices that enclose the reduced data and then transform the reduced data back to the gene expression profiles. The success application of these unsupervised approaches will depend on the availability of large number of observation over a wide range of tissues [14,16]. In addition, these algorithms do not use the biological knowledge on the cell type markers.

Several supervised and semi-supervised computational deconvolution algorithms have also been proposed to tackle this problem [17-21]. However, they require prior knowledge of either the cell type frequencies within a given tissue [19,20], or the in vitro gene expression profiles of each component cell type [17,18]. In reality, this information can be difficult to obtain and presents a major roadblock for these kinds of approaches. Our previous work [22] has proved that gene expression deconvolution should be done in linear space rather than log-transformed space, as is often used in microarray studies. Based on our previous findings, we propose a novel Digital Sorting Algorithm (DSA) that can deconvolve the expression of a tissue into the component profiles of each cell type using a set of marker genes that are highly expressed in each cell type.

## Results and discussion

**Figure 1:**
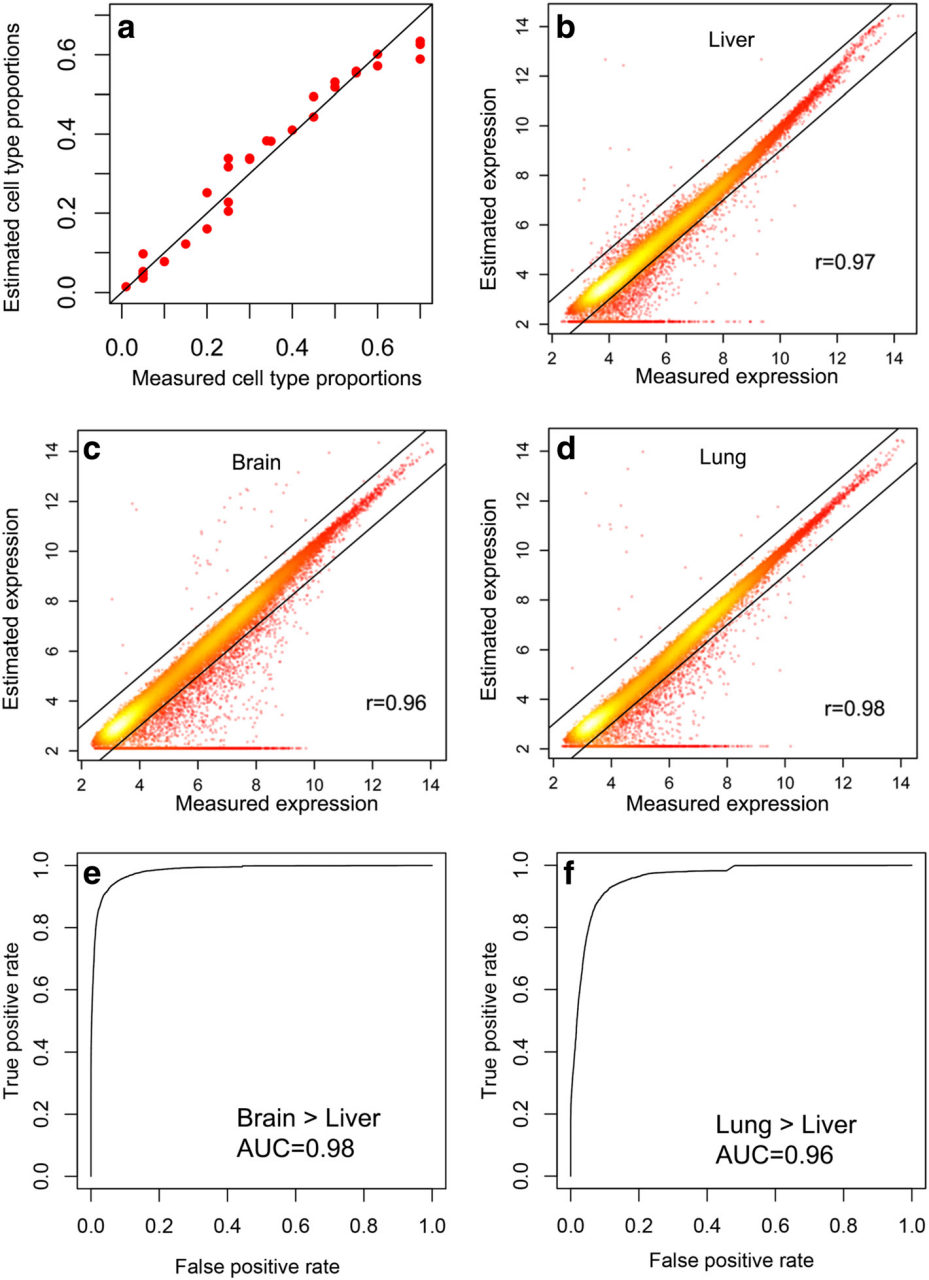
**Unbiased estimation of tissue type specific profiles. (a)** Mixing proportions were estimated using markers for liver, brain and lung. DSA estimation can recapitulate the true percentage of each cell type in the mixture. **(b-d)** DSA estimation of liver, brain and lung gene expression profiles compared against true expression profiles measured using pure tissue samples. **(e-f)** ROC analysis on differential gene expression analysis of brain vs. liver and lung vs. liver using DSA.

To test whether our proposed DSA algorithm can estimate the cell type proportions in a mixed tissue or cell population, we analyzed a benchmark dataset where RNA from the liver, brain and lung of a rat were mixed at 11 different proportions and the mixing parameters are known. The gene expression of pure liver, brain, and lung, and of the mixed samples, was measured using Affymetrix expression arrays. DSA uses a set of gene markers that are highly expressed in specific cell types to estimate the cell type frequencies; the expression level of these markers in pure cell types is not required. A list of tissue-specific markers for the liver, brain and lung was obtained from Tissue-specific Gene Expression and Regulation (TIGER) [23] database and GENENOTE [24] (Additional file 1: Table S1). Using these markers, we were able to estimate the cell type frequencies for each cell type from the mixtures (Figure 1a). Our results demonstrate that DSA can accurately estimate the cell type proportions using marker genes.

Next, we examined whether DSA can accurately deconvolve the gene expression profile from mixed tissue samples into tissue specific expression profiles. Using the cell type frequencies we estimated using marker genes, we were able to accurately estimate the expression profiles of the liver, brain and lung cells that constitute the mixture (Figure 1b-d). The deconvolved expression was highly correlated with the true gene expression profile in each tissue type. The error measure was smaller for genes that are highly expressed, as would be predicted given that technical variations tend to have larger impact for genes that are expressed at low levels. The effect of the number of marker genes used in estimating the proportion of each cell type was also studied. We randomly sampled the marker genes from the TIGER list (Additional file 1: Table S1). In 100 repetitions, we plotted the correlation and mean absolute difference between the estimated and pure cell-specific expressions against the number of markers used. Our results demonstrate that DSA is robust to the number of marker genes and only requires several marker genes for accurate gene expression deconvolution (Additional file 2: Figure S1).

We next asked whether DSA can identify differentially expressed genes between different tissue types. To do this, we computed the gene expression fold change using the deconvolved gene expression profile, and then carried out a Receiver Operator Curve (ROC) analysis to assess DSA’s ability to detect changes more than two-fold between any tissue types. Our results demonstrate that DSA is highly specific and sensitive in identifying differentially expressed genes (Figure 1e-f).

In the benchmark data, liver, brain and lung were used to construct the mixtures. However, the expression differences between different cell types within a tissue sample are much smaller compared to the differences between liver, brain and lung. Hence, we tested whether our DSA algorithm works on real tissue samples composed of cell types with gene expression profiles that are more similar to each other.

**Figure 2:**
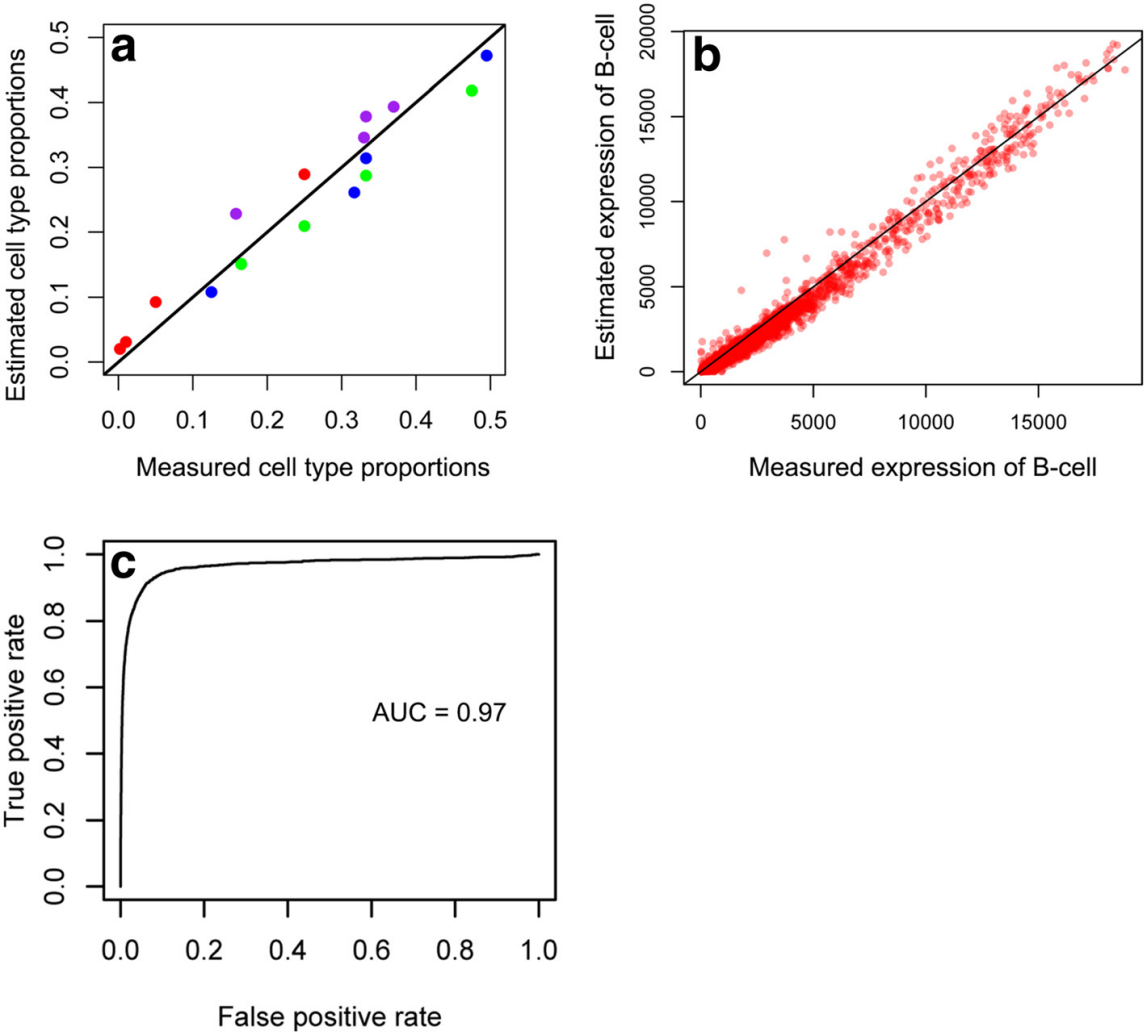
**Unbiased estimation of cell type specific profiles. (a)** Cell type frequencies were estimated using markers for IM-9 cells (green), Raji cells (blue), Jurkat cells (red), and THP-1 cells (purple). **(b)** DSA estimation of the gene expression profiles of IM-9 cells compared against true expression profiles measured using pure cell samples. **(c)** ROC analysis on differential gene expression analysis of estimated IM-9 cells vs. Jurkat cells using DSA.

IM-9, Raji, Jurkat and THP-1 cells were mixed in different proportions and the expression profile of each mixture was measured by microarray [19]. Marker genes for each of the cell types was extracted from the Immunogenetic Related Information Source (IRIS) database [25]. First, we used the genes that are highly expressed in each of these cells (Additional file 3: Table S2) to estimate the cell type proportions accurately (Figure 2a). Next, using the estimated cell type frequencies, we deconvolved the expression profiles of the mixture into profiles for each individual cell type (Figure 2b and Additional file 4: Figure S2). Finally, to test whether the estimated expression profiles of immune cells can be used to identify genes that are differentially expressed between cell types, we applied an ROC analysis on our deconvolved expression profiles (Figure 2c and Additional file 5: Figure S3). High AUC values (0.8 or higher) were observed, indicating that differentially expressed genes can be identified accurately with high specificity and sensitivity.

Population specific expression analysis (PSEA) [26] is an algorithm that has the same input parameters as DSA. However, PSEA uses the marker gene information as normalization factors in the gene expression deconvolution analysis. Hence, the estimated gene expression profiles are not the absolute gene expression values, but are relative to the average of the marker genes for each cell type. In practice, the marker genes from different cells are not guaranteed to have the same expression level. This critical assumption of PSEA makes comparing results between different cell types biased towards the marker gene expression.

**Figure 3:**
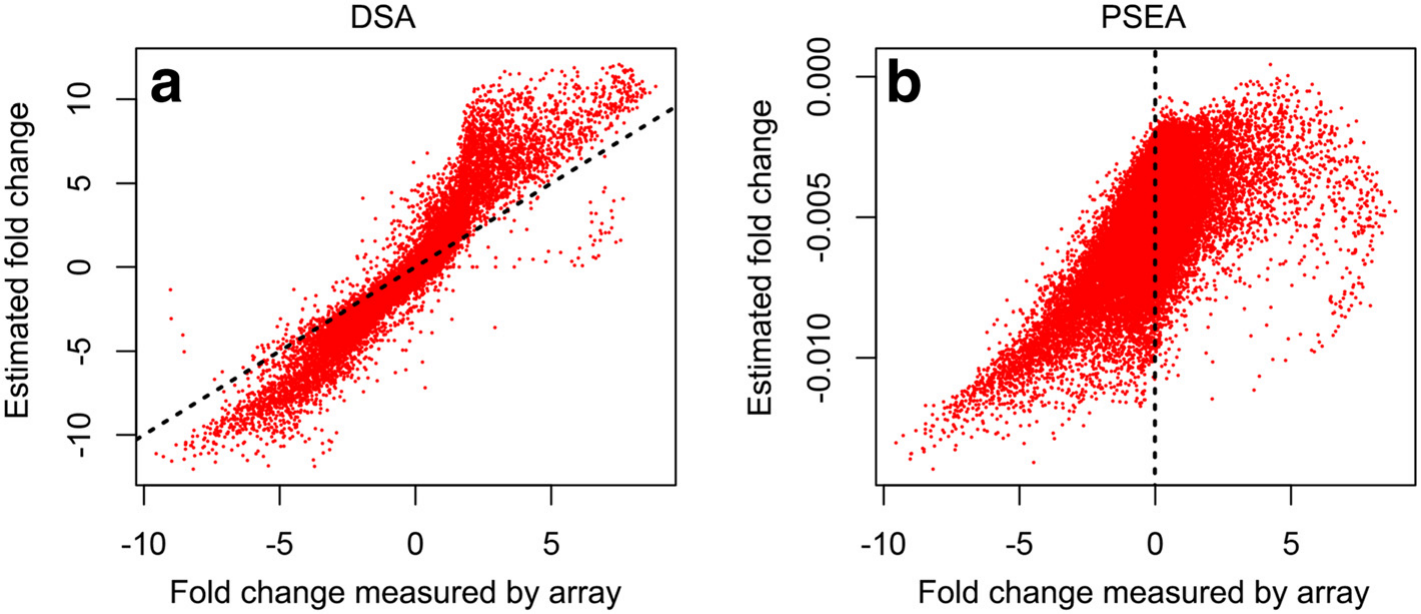
**Comparison between DSA and PSEA. (a)** Fold change estimated by DSA compared against the true fold change between liver and brain samples. The dotted line represents the reference line where all the points should follow. **(b)** Fold change estimated by PSEA compared against the true fold change between liver and brain samples.

To compare the performance of DSA and PSEA, we tested both algorithms on the liver-brain-lung benchmark dataset. The fold change differences estimated by DSA are highly correlated with the true difference (Figure 3a). However, the fold change differences estimated by PSEA between liver and brain are all negative, due to the fact that the marker genes of liver are expressed at higher levels than the markers genes of brain (Figure 3b). Our results demonstrate that the fold change estimated by DSA is more accurate than PSEA. Since DSA only assumes that marker genes are highly expressed and does not require the marker genes to be expressed at same level, DSA tends to estimate the absolute expression level as measured by the array on pure cell populations.

**Figure 4:**
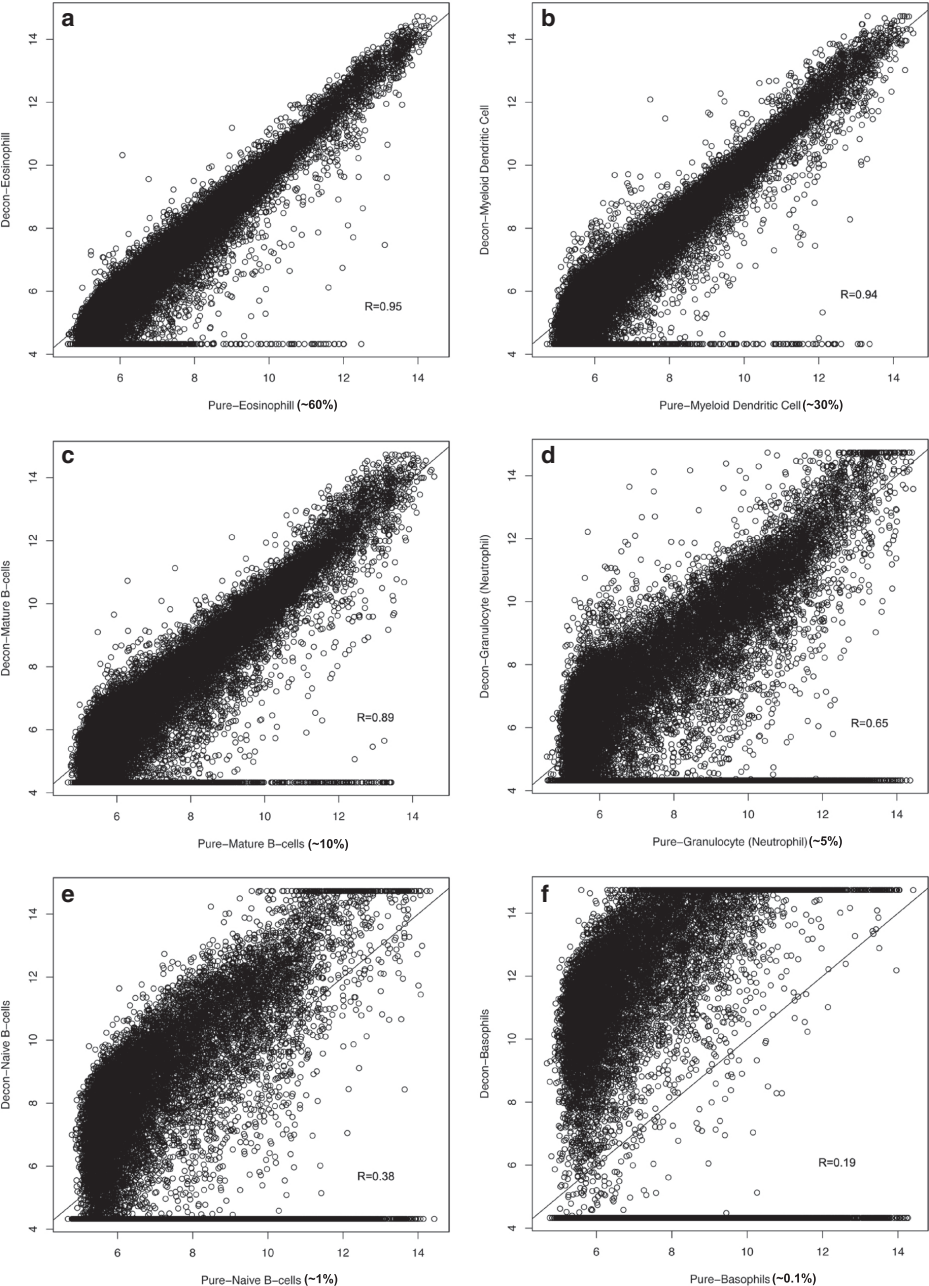
**The estimated transcriptomes for 6 different immune cell types were plotted against the gene expression measured on arrays.** Cell types that have higher percentage in the tissue sample tend to have better estimation accuracy. **(a-f)** Scatter plots of estimated profile against microarray measures in Eosinophil, Myeloid Dendritic, Mature B-cells, Granulocyte, Naïve B-cells, and Basophils.

**Figure 5:**
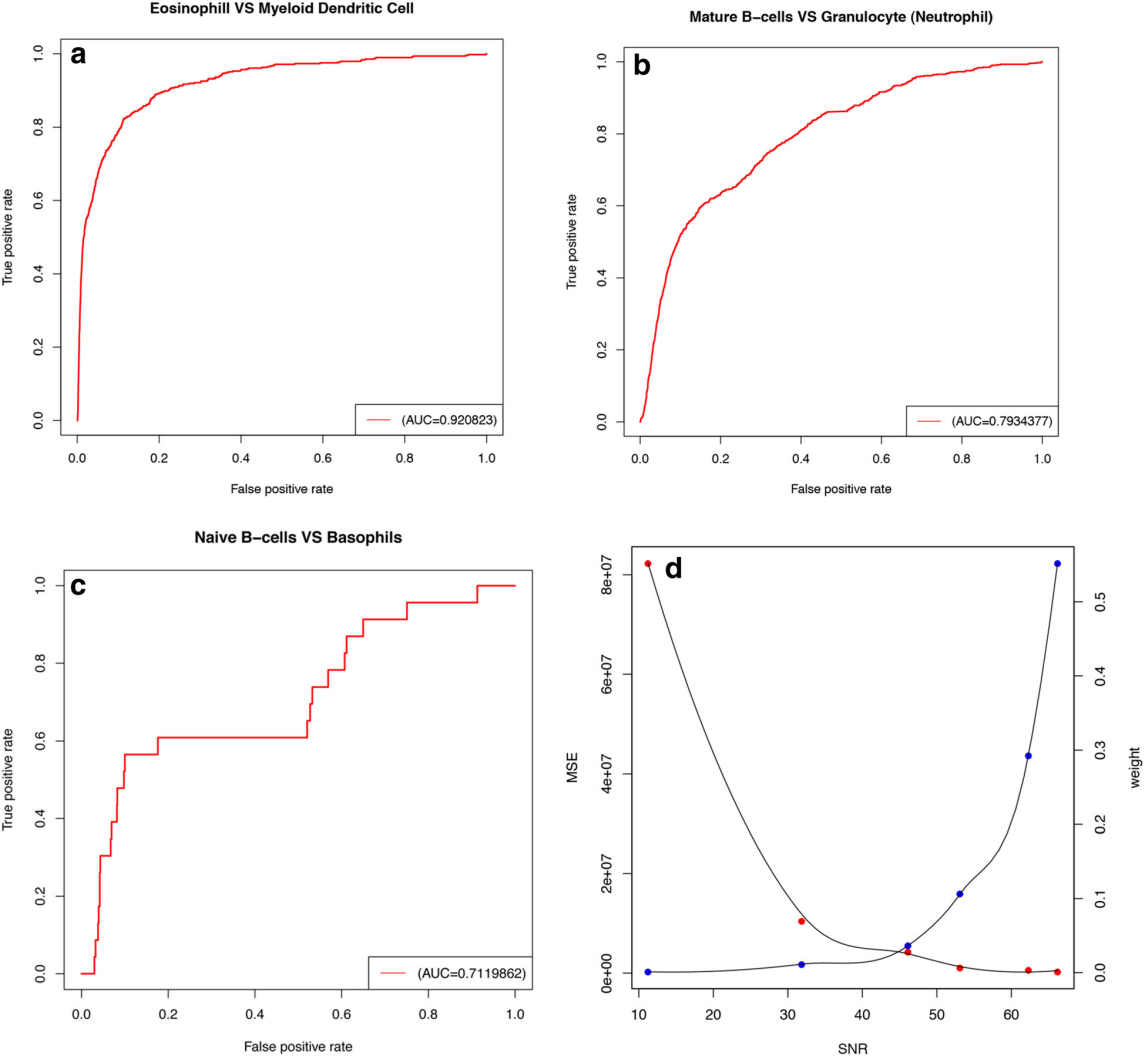
**(a-c) The AUC analysis of cell types that have high and low confidence of deconvolved gene expression profile.** Eosinophil and myeloid dendritic cells have the best AUC scores since these two cell types have the highest proportions in the mixed samples. Naïve B-cells and basophils yield poor but still informative AUC scores, as these two cell types have the lowest frequency in the mixed samples. **(d)** The plot of mean square of error (MSE) and weight against signal-to-noise ratio (SNR). The best cut-off point was observed around 45. Cell types that are present at too low of a frequency in a given tissue will have dramatically increased errors.

We were next interested in determining the lower limit of cell type frequency that could be estimated from a mixed tissue. To test this, we simulated blood samples using 6 different immune cell types with cell type proportions ranging from 60% to 0.1% (Figure 4 and Additional file 6: Table S3). Cells that are present at a frequency greater than 10% in the tissue sample could be accurately estimated (Figure 4a-c). Cell types that are present at greater than 1% but less than 10% can still be estimated, though with relatively larger errors (Figure 4d-e). Cell types that are present at lower than 1% in the tissue failed to be identified (Figure 4f). To summarize our results systematically, we plotted the error of estimation and frequencies of each cell type against the signal to noise ratio (SNR). Clearly, SNR decreases as cell type frequency goes down, and the mean square of errors (MSE) goes up with decreasing SNR (Figure 5d). To assess the sensitivity and specificity of differential gene analysis, we applied ROC analysis on deconvolved expression profiles and found that samples with high accuracy in deconvolution have high AUC value. For samples that have low frequencies in a tissue and poor accuracy in deconvolution, differential gene analysis can still identify genes that are significantly changed between cell types with reasonable -- but significantly reduced -- sensitivity and specificity (Figure 5a-c).

**Figure 6:**
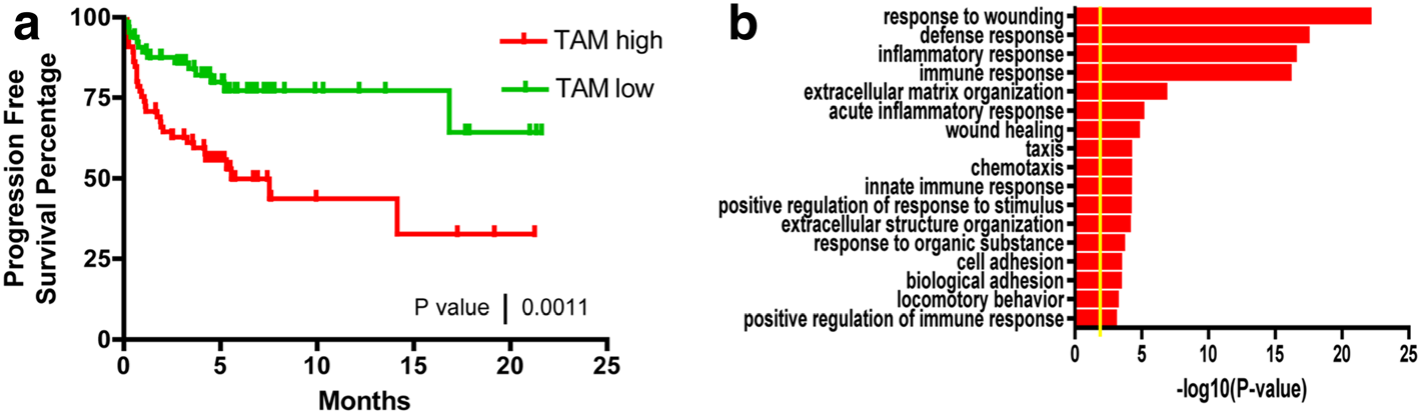
**(a) The percentage of TAMs in Hodgkin’s lymphoma tumors was negatively associated with progression-free survival.** (**b**) Genes that are highly expressed in DSA extracted TAMs are enriched for biological processes characteristic of macrophages, such as response to wounding, immune, inflammatory and defense response.

We next tested whether DSA is capable of estimating the frequency of a cell type in a mixed tissue *in vivo*, and subsequently obtaining its gene expression profile. To this end, we applied our algorithm on a set of human Hodgkin’s lymphomas, in which macrophage frequency was shown to predict progression-free survival [8]. A set of tumor associated macrophage (TAM) specific genes was selected by comparing mouse mammary tumor associated macrophages to normal mouse splenic macrophages [9] (Additional file 7: Table S4). Using these TAM-specific genes, DSA was applied to the gene expression profiles of a set of Hodgkin’s lymphomas. For each tumor, DSA was able to estimate the percentage of TAMs in the sample. We found that the estimated TAM percentage is a predictor of progression free survival for these patients (Figure 6a). A 30-fold lower p-value was achieved using our estimation than using CD68 as a marker for TAMs. The hazard ratio between TAM high and TAM low group is 2.7. Using DSA, we were able to obtain the gene expression profile of TAMs in the patient samples. By comparing the TAM transcriptome to the tumor transcriptome, we identified a list of genes that are highly expressed in TAMs. When Gene Ontology (GO) analysis was applied to these genes [10], we identified response to wounding, defense response, and inflammatory response as high significantly enriched biological processes (Figure 6b). These results confirmed that the estimated gene expression profile is indeed enriched for macrophage related functions.

## Conclusions

In conclusion, we have demonstrated the general feasibility of a Digital Sorting Algorithm (DSA) to obtain cell type specific gene expression profiles from a complex tissue. DSA represents a dramatic improvement over the conventional deconvolution approaches, which typically require prior knowledge of cell type frequencies or in vitro gene expression profiles for each cell type. By using cell type specific genes, DSA overcomes these limitations. We have also demonstrated that DSA is an unbiased estimation algorithm for signal reconstruction and deconvolution. Downstream analysis, such as differential gene analysis, will benefit from digital sorting and yield better results. Most important of all, we have demonstrated that DSA can be used to extract the expression profile of a specific cell type from a complex tissue. This will allow for investigation of the properties of specific cell types in mixed tissues *in vivo*. For example, we can obtain the gene expression profile of a particular cell type in the cancer microenvironment just from microarray data from the bulk tumor. In principle, the DSA framework could be applied to any unbiased high-throughput dataset, such as global DNA methylation array, next generation sequencing data, metabolic data, and proteomics. Partially, RNA-seq is a more accurate technology compared to microarray. The linearity assumption holds true in RNA-seq studies, hence we believe that our DSA framework can also be applied to RNA-seq data.

In many real-world applications, a small number of cell type specific markers are often available to molecular biologists since these markers are frequently used in biochemical assays. For example, the cancer stem cell markers are known for many types of tumors. The use of these markers in gene expression deconvolution greatly improved the performance and also enabled the application of this algorithm not only to cancer studies, but also to other biological studies involved with heterogeneous samples.

DSA is implemented in a single R package (https://github.com/zhandong/DSA). The package also includes sample data from liver, brain and lung benchmark data and the cell type specific markers.

## Methods

### Microarray analysis

Liver, brain and lung tissues derived from a single rat were homogenized, extracted, and mixed in 11 different proportions in triplicates. The gene expression profile of these mixed tissues were measured using Affymetrix array and can be obtained from GSE19830. Immune cell expression profiles were obtained from GSE11057 and GSE24759. Hodgkin’s lymphoma dataset was obtained from GSE17920. The tumor associated macrophage marker genes were obtained from GSE18404.

### Simulated immune cells

Six different immune cell lines that were used to construct references are available in the simulated blood samples. The weights were sampled randomly in decreasing order (Additional file 6: Table S3).

### Linear model on gene expression deconvolution

Let *S* be an *n* × *k* gene expression matrix that contains *k* cell types and *n* genes, *W* be a *k* × *p* matrix where each column of *W* contains the frequencies of *k* cell types in a particular observation, and *O* be an *n* × *p* expression matrix that contains the observed gene expression level, where *n* represents the number of genes and *p* is the number of observed tissue samples. The mixing process can be modeled through a linear model:

(1) O=S×W

where *S* represents the source signal, *W* is the weight matrix for cell type frequencies, and *O* is the observation on tissue samples. In a typical gene expression profiling setting, *O* is often measured through microarray or RNA-seq. Both *W* and *S* are unknown and our goal is to estimate *S*. We approach this problem by first estimating *W* using cell type specific markers and then solve the linear model using estimated *W*.

### Estimate cell type frequencies matrix *W* from marker genes

Given that we know a set of genes that has high expression level in a specific cell type and low expression in all other cell types, we can predict the proportion of each cell type present in the tissue sample using these genes. Let *X*_*S*_ be an *m* × *k* matrix that contains *m* cell type specific genes for *k* cell types. For each cell type, there could be multiple cell type specific genes. Since each gene is highly expressed in a single cell type, we can take an average of all the genes that are highly expressed in a single cell type and save the matrix as ${\tilde{X}}_{S}$.

$$\begin{array}{l} X_{S}=\left(\begin{array}{l} \begin{array}{cccc} g_{11} & \;0 & \;\ldots & 0 \end{array}\\ \begin{array}{cccc} g_{21} & 0 & \ldots & 0\\ 0 & g_{32} & \ldots & 0\\ 0 & g_{42} & \ldots & 0\\ 0 & g_{52} & \ldots & 0 \end{array}\\ \begin{array}{cccc} \;0 & \;0 & \;\ddots & \vdots \end{array}\\ \begin{array}{cccc} \;0 & \;0 & \;\ldots & g_{\mathrm{mk}} \end{array} \end{array}\right)\Rightarrow \\ {\tilde{X}}_{S}=\left(\begin{array}{cccc} {\bar{g}}_{1} & 0 & \ldots & 0\\ 0 & {\bar{g}}_{2} & \ldots & 0\\ 0 & 0 & \ddots & 0\\ 0 & 0 & \ldots & {\bar{g}}_{k} \end{array}\right) \end{array}$$

Although ${\tilde{X}}_{S}$ is unknown, the corresponding gene expression for cell type specific markers, *O*_*S*_ and *Õ*_*S*_ are measured on the observed mixed samples. Substitute ${\tilde{X}}_{S}$ and *Õ*_*S*_ to equation (1), we obtain

(2) $${\tilde{O}}_{s}={\tilde{X}}_{S}\times W$$

Since ${\tilde{X}}_{S}$ is a diagonal matrix, we can multiply each side of equation (2) by the ${\tilde{X}}_{S}^{-1}$ and obtain

(3) $${\tilde{X}}_{S}^{-1}{\tilde{O}}_{s}=W$$

Given that *W* is the frequency matrix and each column of *W* sums to 1 [15], we can form a system of linear questions of *k* unknown parameters, ${\bar{g}}_{1}\ldots {\bar{g}}_{k}$, where each column of

(4) $$\sum_{i=1}^{k}{\left({\tilde{X}}_{S}^{-1}{\tilde{O}}_{s}\right)}_{\mathrm{ij}}=1$$

When the number of observations on the mixed samples is greater the number of cell types involved that is *p* >*k*, we can solve the system of equations with *k* unknown parameters. Once ${\bar{g}}_{1}\ldots {\bar{g}}_{k}$ is known, we can take ${\tilde{X}}_{S}^{-1}$ into equation (3) and compute the cell type frequency matrix.

### Digital sorting on tissue samples

Input: Expression data on tissue samples and a set of gene symbols that is known to be highly expressed in a specific cell type.

Output: Expression profile for each of the cell types in a tissue.

Step I: If *W* is known, proceed to step II, else estimate *W* using *X*_*S*_ and equation 3.

Step II: Estimate *S* through quadratic programming.

$$\begin{array}{c} \min_{S}\|O-\mathrm{SW}\|{}_{2}\\ s.t\;S≺t_{1}\;\mathrm{and}\;S≻t_{2} \end{array}$$

where *O* is the gene expression profile on tissue samples, *S* is the expression profile for pure cell types, *W* is the weight matrix estimated using the marker genes, and *t*_*1*_ and *t*_*2*_ is the maximum and minimum measurable gene expression level. R package ‘*quadprog*’ is used to solve the quadratic programming problem.

### Receiver operator characteristic (ROC) analysis

R package ‘*ROCR*’ from CRAN [27] was used to compute the ROC curve and the area under curve (AUC). Specifically, genes with more than 2 fold increase or decrease are included in the reference list as the positive set. Our goal is to assess the true positive rate and false positive rate in identifying these genes using our estimated gene expression profiles. A ratio between the estimated gene expression profiles of two different cell types is used to compute the ROC curve. A gene is classified into the positive set if the ratio of this gene is greater than a threshold *t*. A ROC curve is generated by varying *t*.

### Survival analysis

Five tumor associated macrophage (TAM) marker genes were selected by comparing the macrophage in mouse mammary tumor to the normal splenic macrophages. The percentage and expression profile of TAMs were estimated using DSA algorithm. A cox proportional hazard model was used and a significant association was identified with survival. Further, patients are dichotomized into two groups by comparing to the median percentage of TAMs. A log rank test was calculated on these data.

## Competing interests

The authors declare no competing interests.

## Authors’ contributions

YZ, YWW, and KP performed the analysis of the data. YZ and YW developed the software. LMLC and ZL conceived and designed the study and wrote the manuscript. All authors read and approved the final manuscript.

## Supplementary Material

Additional file 1: Table S1 Marker genes for liver, brain and lung Click here for file

Additional file 2: Figure S1 Correlation and mean absolute difference between DSA estimation and original cell specific expression using various number of marker genes. The experiment was repeated 100 times on each number of marker genes. Result show that DSA was robust to the number of marker genes used, even with small number of marker genes. Click here for file

Additional file 3: Table S2 Marker genes for cells of the immune system. Click here for file

Additional file 4: Figure S2 DSA estimation of T-cells, B-cells, and monocytes. Cell type specific markers were extracted from Immune Response In Silico database. Using these markers, DSA was able to faithfully identify the gene expression profile of B-cells, T-cells, and monocytes from mixture samples. Click here for file

Additional file 5: Figure S3 AUC analysis for differential gene analysis. Differential gene expression analysis using estimated pure cell gene expression profiles was able to accurately identify genes that are differentially expressed between different cell types. Click here for file

Additional file 6: Table S3 Cell type proportions for simulated blood samples. Click here for file

Additional file 7: Table S4 Marker genes for Macrophages. Click here for file

## References

[B1] Cobb JP Application of genome-wide expression analysis to human health and disease Proc Natl Acad Sci 2005 102 4801 4806 10.1073/pnas.0409768102 15781863 PMC555033

[B2] Repsilber D Kern S Telaar A Walzl G Black GF Selbig J Parida SK Kaufmann SH Jacobsen M Biomarker discovery in heterogeneous tissue samples -taking the in-silico deconfounding approach BMC Bioinformatics 2010 11 27 10.1186/1471-2105-11-27 20070912 PMC3098067

[B3] Palmer C Diehn M Alizadeh AA Brown PO BMC Genomics 2006 7 115 10.1186/1471-2164-7-115 16704732 PMC1479811

[B4] Hanahan D Weinberg RA Hallmarks of cancer: the next generation Cell 2011 144 646 674 10.1016/j.cell.2011.02.013 21376230

[B5] Yang F Stromal expression of connective tissue growth factor promotes angiogenesis and prostate cancer tumorigenesis Cancer Res 2005 65 8887 8895 10.1158/0008-5472.CAN-05-1702 16204060

[B6] Tuxhorn JA Ayala GE Smith MJ Smith VC Dang TD Rowley DR Reactive stroma in human prostate cancer: induction of myofibroblast phenotype and extracellular matrix remodeling Clin Cancer Res 2002 8 2912 2923 12231536

[B7] Joyce JA Pollard JW Microenvironmental regulation of metastasis Nat Rev Cancer 2008 9 239 252 10.1038/nrg2317 19279573 PMC3251309

[B8] Yu H Kortylewski M Pardoll D Crosstalk between cancer and immune cells: role of STAT3 in the tumour microenvironment Nat Rev Immunol 2007 7 41 51 10.1038/nri1995 17186030

[B9] Salsman VS Chow KKH Shaffer DR Kadikoy H Li X-N Gerken C Perlaky L Metelitsa LS Gao X Bhattacharjee M Hirschi K Heslop HE Gottschalk S Ahmed N Crosstalk between medulloblastoma cells and endothelium triggers a strong chemotactic signal recruiting T lymphocytes to the tumor microenvironment PLoS One 2011 6 e20267 10.1371/journal.pone.0020267 21647415 PMC3103535

[B10] Qian B-Z Li J Zhang H Kitamura T Zhang J Campion LR Kaiser EA Snyder LA Pollard JW CCL2 recruits inflammatory monocytes to facilitate breast-tumour metastasis Nature 2011 475 222 225 10.1038/nature10138 21654748 PMC3208506

[B11] Pahler JC Tazzyman S Erez N Chen Y-Y Murdoch C Nozawa H Lewis CE Hanahan D Plasticity in tumor-promoting inflammation: impairment of macrophage recruitment evokes a compensatory neutrophil response Neoplasia (New York, NY) 2008 10 329 340 10.1593/neo.07871 PMC2288539 18392134

[B12] Okaty BW Sugino K Nelson SB A quantitative comparison of cell-type-specific microarray gene expression profiling methods in the mouse brain PLoS One 2011 6 e16493 10.1371/journal.pone.0016493 21304595 PMC3029380

[B13] Hoelzinger DB Nakada M Demuth T Rosensteel T Reavie LB Berens ME Autotaxin: a secreted autocrine/paracrine factor that promotes glioma invasion J Neurooncol 2007 86 297 309 17928955 10.1007/s11060-007-9480-6

[B14] Quon G Morris Q ISOLATE: a computational strategy for identifying the primary origin of cancers using high-throughput sequencing Bioinformatics 2009 25 2882 2889 10.1093/bioinformatics/btp378 19542156 PMC2781747

[B15] Schwartz R Shackney SE Applying unmixing to gene expression data for tumor phylogeny inference BMC Bioinformatics 2010 11 42 10.1186/1471-2105-11-42 20089185 PMC2823708

[B16] Qiao W Quon G Csaszar E Yu M Morris Q Zandstra PW PERT: a method for expression deconvolution of human blood samples from varied microenvironmental and developmental conditions PLoS Comput Biol 2012 8 e1002838 10.1371/journal.pcbi.1002838 23284283 PMC3527275

[B17] Lu P Expression deconvolution: a reinterpretation of DNA microarray data reveals dynamic changes in cell populations Proc Natl Acad Sci 2003 100 10370 10375 10.1073/pnas.1832361100 12934019 PMC193568

[B18] Wang M Master SR Chodosh LA BMC Bioinformatics 2006 7 328 10.1186/1471-2105-7-328 16817968 PMC1559723

[B19] Abbas AR Wolslegel K Seshasayee D Modrusan Z Clark HF Deconvolution of blood microarray data identifies cellular activation patterns in systemic lupus erythematosus PLoS One 2009 4 e6098 10.1371/journal.pone.0006098 19568420 PMC2699551

[B20] Shen-Orr SS Tibshirani R Khatri P Bodian DL Staedtler F Perry NM Hastie T Sarwal MM Davis MM Butte AJ Cell type-specific gene expression differences in complex tissues Nat Methods 2010 7 287 289 10.1038/nmeth.1439 20208531 PMC3699332

[B21] Gaujoux R Seoighe C Semi-supervised nonnegative matrix factorization for gene expression deconvolution: a case study Infect Genet Evol 2012 12 913 921 10.1016/j.meegid.2011.08.014 21930246

[B22] Zhong Y Liu Z Gene expression deconvolution in linear space Nat Methods 2012 9 8 9 author reply 9 22205510 10.1038/nmeth.1830

[B23] Liu X Yu X Zack DJ Zhu H Qian J TiGER: a database for tissue-specific gene expression and regulation BMC Bioinformatics 2008 9 271 10.1186/1471-2105-9-271 18541026 PMC2438328

[B24] Yanai I Benjamin H Shmoish M Chalifa-Caspi V Shklar M Ophir R Bar-Even A Horn-Saban S Safran M Domany E Lancet D Shmueli O Genome-wide midrange transcription profiles reveal expression level relationships in human tissue specification Bioinformatics 2005 21 650 659 10.1093/bioinformatics/bti042 15388519

[B25] Kelley J de Bono B Trowsdale J IRIS: a database surveying known human immune system genes Genomics 2005 85 503 511 10.1016/j.ygeno.2005.01.009 15780753

[B26] Kuhn A Thu D Waldvogel HJ Faull RLM Luthi-Carter R Population-specific expression analysis (PSEA) reveals molecular changes in diseased brain Nat Methods 2011 8 945 947 10.1038/nmeth.1710 21983921

[B27] Sing T Sander O Beerenwinkel N Lengauer T ROCR: visualizing classifier performance in R Bioinformatics 2005 21 3940 3941 10.1093/bioinformatics/bti623 16096348

