# Supplementary material for: Digital sorting of complex tissues for cell type-specific gene expression profiles
Source: BMC Bioinformatics. 2013 Mar 7;14:89. doi: 10.1186/1471-2105-14-89 (PMC3626856; doi:10.1186/1471-2105-14-89)
Supplement: Additional file 2: Figure S1 — Correlation and mean absolute difference between DSA estimation and original cell specific expression using various number of marker genes. The experiment was repeated 100 times on each number of marker genes. Result show that DSA was robust to the number of marker genes used, even with small number of marker genes. [file 1471-2105-14-89-S2.pdf]

Supplementary Figure 1

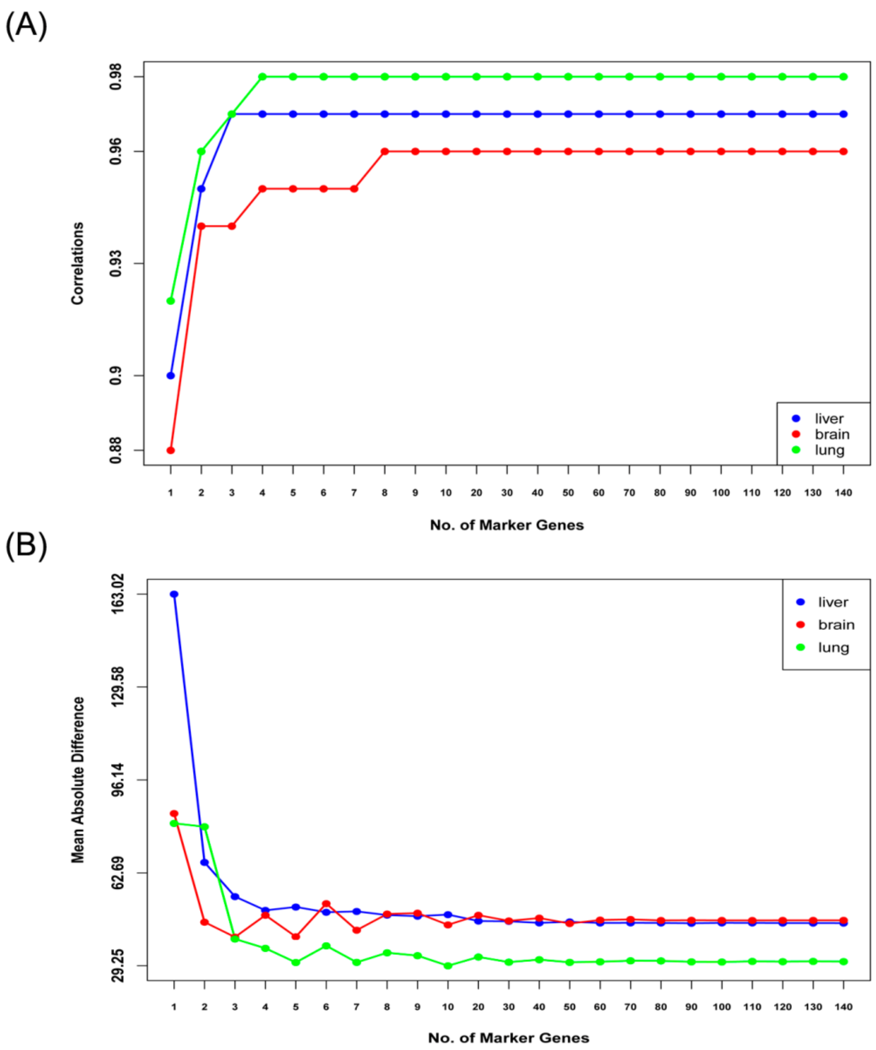

Marker genes were randomly selected from the TIGER list at different sizes, for 100 times on each size. In each repetition, the estimated weight matrix using the sampled marker genes was then used to estimate the cell-type specific signals. Correlation and mean absolute difference (MAD) between the original and estimated signals were measured. (A) shows the average correlation and (B) shows the average MAD from the 100 iterations using different number of marker genes.
