# Supplementary figures and images for: Digital sorting of complex tissues for cell type-specific gene expression profiles
Source: BMC Bioinformatics. 2013 Mar 7;14:89. doi: 10.1186/1471-2105-14-89 (PMC3626856; doi:10.1186/1471-2105-14-89)

Supplementary Figure 2

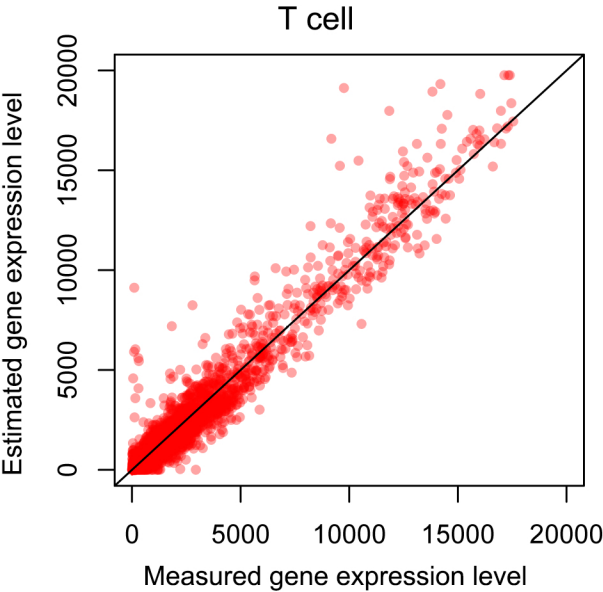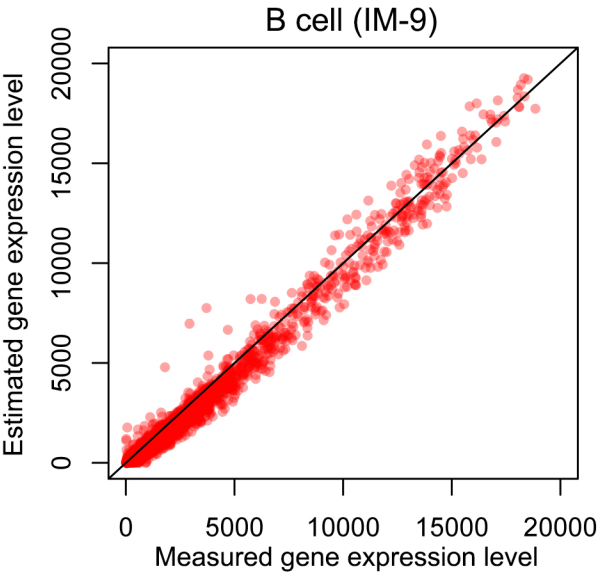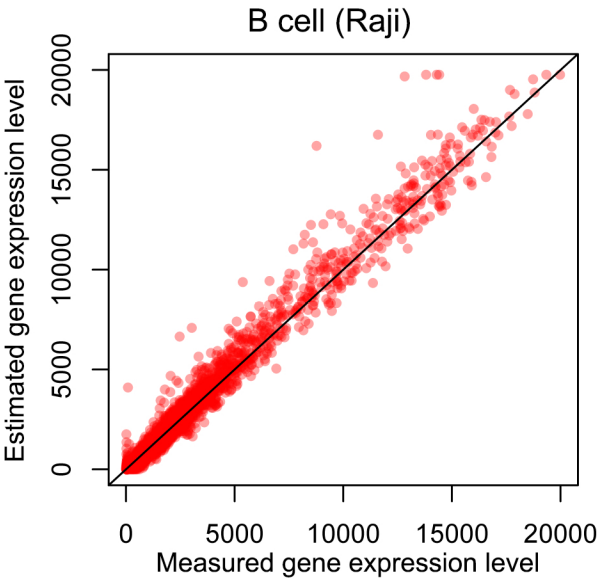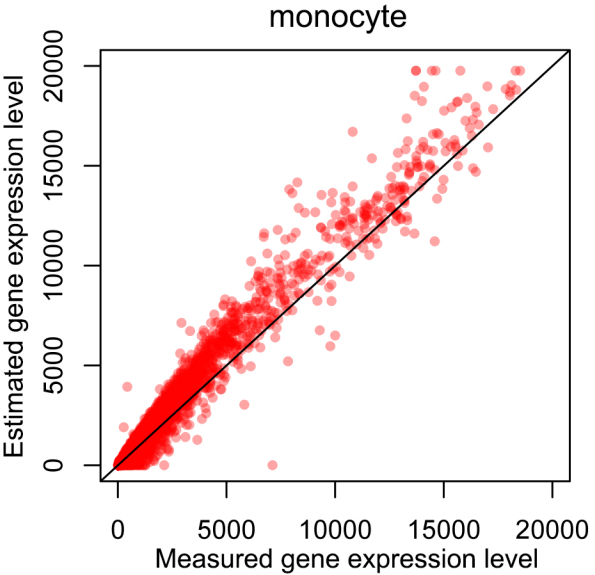

Supplement: Additional file 4: Figure S2 — DSA estimation of T-cells, B-cells, and monocytes. Cell type specific markers were extracted from Immune Response In Silico database. Using these markers, DSA was able to faithfully identify the gene expression profile of B-cells, T-cells, and monocytes from mixture samples. [file 1471-2105-14-89-S4.pdf]

Supplementary Figure 3

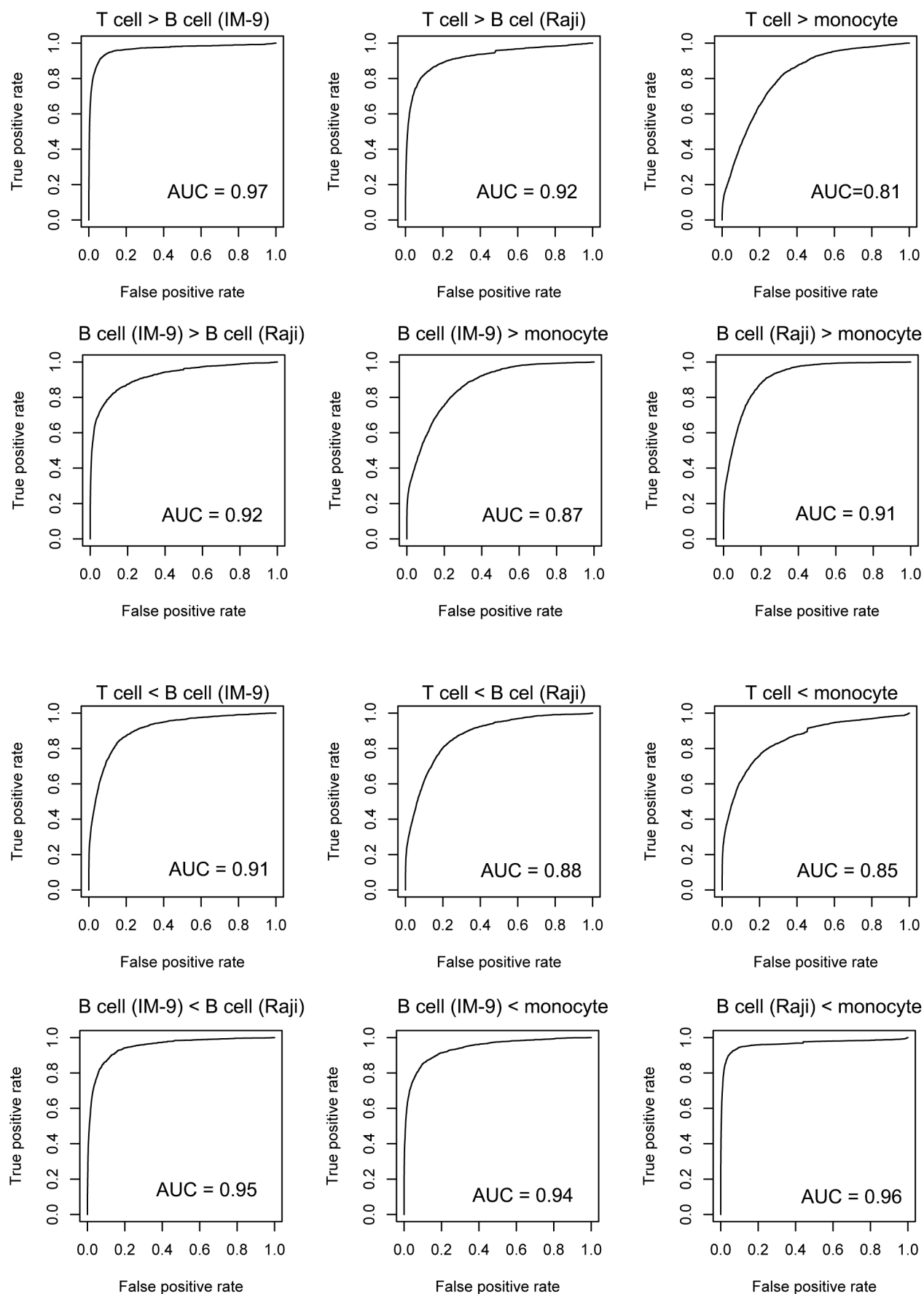

Supplement: Additional file 5: Figure S3 — AUC analysis for differential gene analysis. Differential gene expression analysis using estimated pure cell gene expression profiles was able to accurately identify genes that are differentially expressed between different cell types. [file 1471-2105-14-89-S5.pdf]
